# Association between serum uric acid and phase angle in patients with type 2 diabetes mellitus: A cross-sectional study

**DOI:** 10.3389/fendo.2023.1124565

**Published:** 2023-03-27

**Authors:** Yezi Hu, Jie Liu, Hui Jin

**Affiliations:** ^1^ Department of Clinical Nutrition, Affiliated Zhongda Hospital of Southeast University, Nanjing, China; ^2^ Department of Vascular and Endovascular Surgery, Chinese PLA General Hospital, Beijing, China

**Keywords:** serum uric acid, phase angle, type 2 diabetes mellitus, nutritional status, body composition

## Abstract

**Background:**

The purpose of this analysis was to investigate the associations between serum uric acid and phase angle in patients with type 2 diabetes mellitus.

**Methods:**

In this retrospective cross-sectional study, we included 200 type 2 diabetes mellitus (T2DM) patients treated during 2018–2019 at Zhongda Hospital Southeast University. Phase angle (PhA) and other body composition indicators were measured by bioelectrical impedance analysis (BIA). All patients underwent routine clinical examinations on the day of hospitalization, and the basic information and clinical symptoms of these patients were recorded.

**Results:**

Serum uric acid (UA) was significantly associated with PhA (p <0.001). Overall, in the crude model and minor, all adjusted models (crude model, Models I–II), the phase angle increased as the tertiles of serum uric acid increased. In the minor adjusted model (Model I, adjustment for age and duration) fully adjusted model (Model II, adjustment for age, duration, Lpa, BMI, and WHR), the adjusted β for participants in tertiles of serum uric acid were 0.26 (95% CI: 0.05–0.46) and 0.32 (95% CI: 0.11–0.54), respectively, compared with those in the lowest tertile 1.

**Conclusion:**

There was a nonlinear relationship between serum uric acid and PhA in T2DM patients, and the phase angle increased as uric acid increased within a certain range, and this effect disappeared when uric acid exceeded a certain value.

## 1 Introduction

Phase angle (PhA) is a useful instrument to identify dysfunction in cell membrane integrity; therefore, it is seen as an important prognostic marker in many clinical contexts (1), such as malnutrition (2). PhA is also used as an important nutritional indicator. In general, the lower the PhA, the worse the nutrition outcome. According to several studies, people with diabetes show decreased PhA compared with healthy people (3, 4). In addition, PhA is also directly associated with other nutritional markers such as BMI and grip strength (5). Measuring PhA is relatively cheap and is considered a non-invasive procedure based on bioelectrical impedance (BIA) (6). Many recent studies have shown that bioimpedance measurements such as visceral fat area can be correlated with nutritional indicators (7).

Uric acid is the end product of purine metabolism and is considered abnormally high in the human body when its serum concentration exceeds 360 or 420 mmol/L (8). Uric acid may have both proinflammatory and antioxidant properties, and therefore its exact role in disease risk is not clear (9). Studies have shown that SUA may be a nutritional marker in hemodialysis patients (10). In recent years, there have been many studies on UA in the kidney diseases field. However, in reviewing the literature, we did not find any information regarding the association between UA and nutritional status in diabetes mellitus patients. As far as we know, there is no information in the literature to explore the association between serum uric acid and phase angle in patients with diabetes mellitus. The relationship between phase angle and metabolism in diabetic patients has been worked out in many laboratories, and it is well reported. However, reports on direct work related to uric acid and phase angle in relation to diabetes are few (3, 11, 12).

We therefore aimed to investigate the associations between serum uric acid and phase angle in patients with diabetes mellitus. Paying attention to the relationship between serum uric acid and nutritional status in diabetes mellitus patients has predictive value for early intervention in malnutrition.

## 2 Methods

### 2.1 Study design and participants

**Figure 1:**
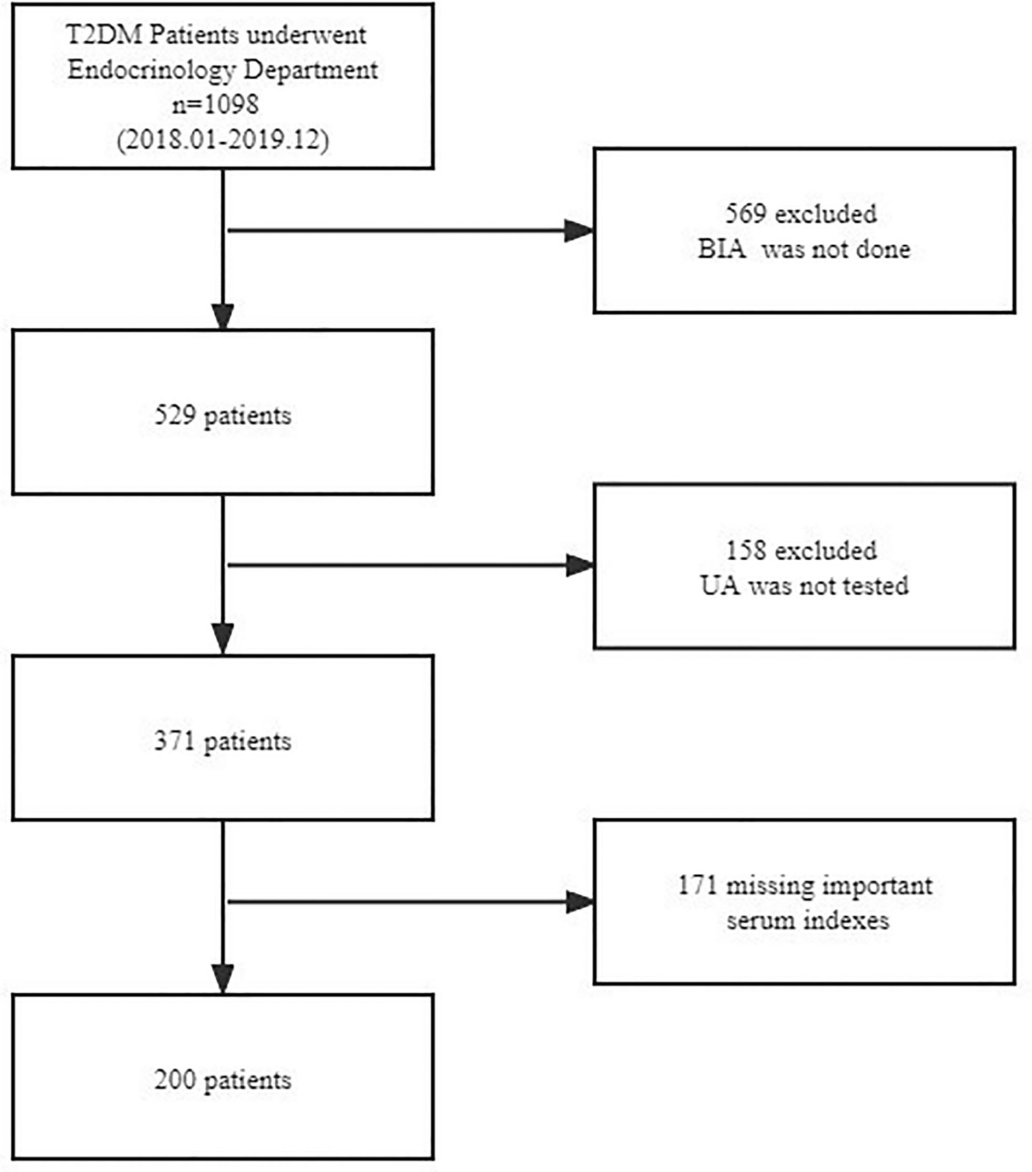
Flowchart of participant selection. T2DM, type 2 diabetes mellitus; BIA, bioelectrical impedance analysis; UA, uric acid.

A total of 200 patients with T2DM who were admitted to the Department of Endocrinology and Metabolism at Zhongda Hospital Southeast University between January 2018 and December 2019 were recruited in this cross-sectional study. The flow chart displaying patient selection is presented in **Figure 1**. Inclusion criteria for the T2DM group include having been previously diagnosed with T2DM according to American Diabetes Association (ADA) criteria. Children were diagnosed when their fasting plasma glucose was ≥ 126 mg/dl (7.0 mmol/L). Exclusion criteria were: 1) age <18 years; 2) severe liver and kidney functional abnormalities; 3) malignant tumor; 4) the use of drugs that affect uric acid metabolism; and 5) an incomplete laboratory examination or BIA data.

The study adhered to the principles of the Declaration of Helsinki and was approved by the Ethics Review Board of Zhongda Hospital Southeast University. Given the retrospective nature of the study and the use of anonymized patient data, the requirements for informed consent were waived.

### 2.2 Measurement body composition and UA

Body composition was measured using multi-frequency BIA (InBody 770; InBody Co., Ltd., Korea) in the morning on the patient’s first day in hospital. Before the BIA test, patients were required to abstain from food and water, empty their bladders, remove their shoes and socks, wear clothing of known weight, and remove other items. The participants were instructed to stand barefoot on the metal footplate of the analyzer, holding the handgrip with arms straight and pointing downwards in a neutral standing position. The surface of the hand electrode was placed in contact with each of the participant’s five fingers, whereas the participant’s heels and forefoot were placed on the circular-shaped foot electrode. All parameters, including PhA, waist–hip ratio (WHR), skeletal muscle mass (SMM), and body mass index (BMI), were directly measured and recorded by the devices. After 8–10 h of fasting, blood samples were collected for measurement of uric acid, fasting plasma lipids, glucose, and HbA1c.

### 2.3 Statistical analysis

Patient characteristics were analyzed according to serum uric acid tertiles. Categorical variables are expressed in numbers and percentages. Continuous variables are expressed as mean and standard deviation (SD) for normal distributions or median and interquartile range (IQR) for skewed distributions. We used the chi-square test and Kruskal–Wallis test for the comparison of categorical, normally distributed, and nonnormally distributed continuous variables, respectively.

Univariate linear regression analyses and multivariable linear regression analyses were performed to evaluate the associations between serum uric acid and PhA. According to the recommendation of the Strengthening the Reporting of OBservational studies in Epidemiology (STROBE) statement (13), analyses were first performed without adjustment. Further analyses cumulatively included adjustment for age, duration (a minimally adjusted model), Lpa, BMI, and WHR (a fully adjusted model). Linear regression models were used for the subgroup analyses and included terms for sex group, BMI group, age group, and the interaction of each subgroup.

All the analyses were performed with the statistical software packages R (http://www.R-project.org, The R Foundation) and Free Statistics software version 1.7.1. A two-sided *P*-value of <0.05 was statistically significant.

## 3 Results

### 3.1 Participants selection

Baseline characteristics of the 200 enrolled participants (122 men and 78 women) stratified by serum uric acid level are shown in **Table 1**. Patients enrolled were grouped by the tertiles of serum uric acid levels as follows: UA T1group, ≥196.5 to ≤279 ummol/L; UA T2group, ≥281 to ≤350 ummol/L; and UA T3group, ≥352 to ≤626 ummol/L. Some differences existed between the serum uric acid level groups with respect to various covariates (TC, FPG, FCP, SMM, BMI, WHR, PhA, and TG). The number of patients in thethree groups was 66, 67, and 67, respectively. 

**Table 1:** Clinical characteristics of the study population by serum uric acid levels.

	Serum uric acid			
Characteristic	UA T1 (196.5–279) (n = 66)	UA T2 (281–350) (n = 67)	UA T3 (352–626) (n = 67)	*p*-Value
Age (years)	57.8 ± 12.7	56.7 ± 14.0	52.9 ± 14.9	0.102
Duration (years)	8.9 ± 6.5	8.5 ± 6.4	8.8 ± 8.0	0.922
TC (mmol/L)	4.6 ± 1.1	4.5 ± 1.2	5.1 ± 1.3	0.006
HbA1c (%)	9.4 ± 1.8	9.1 ± 2.0	8.9 ± 1.7	0.279
FPG (mmol/L)	7.1 ± 1.6	7.2 ± 1.6	7.8 ± 1.6	0.045
FCP (nmol/L	0.5 ± 0.3	0.6 ± 0.4	0.8 ± 0.4	< 0.001
SMM (kg)	25.4 ± 5.3	28.3 ± 5.5	30.1 ± 5.6	< 0.001
BMI (kg/m2)	24.0 ± 3.1	25.4 ± 3.2	26.5 ± 3.6	< 0.001
PBF (%)	28.9 ± 7.6	28.6 ± 6.3	28.9 ± 7.6	0.970
WHR	0.9 ± 0.1	0.9 ± 0.1	0.9 ± 0.1	0.009
VFA (cm2)	92.5 ± 36.5	98.0 ± 32.2	106.1 ± 42.3	0.109
Phase Angle (°)	4.9 ± 0.7	5.2 ± 0.7	5.4 ± 0.7	< 0.001
TG (mmol/L)	1.3 (0.9, 2.0)	1.7 (1.0, 2.6)	2.1 (1.4, 4.2)	< 0.001
Lpa (g/L)	112.5 (44.5, 368.5)	160.0 (32.0, 270.5)	60.0 (32.0, 172.5)	0.062

T, tertile; TC, total cholesterol; HbA1c, glycosylated hemoglobin; FPG, fasting plasma glucose; FCP, fasting C-peptide; SMM, skeletal muscle mass; BMI, body mass index; PBF, percent body fat; WHR, waist-to-hip ratio; VFA, visceral fat area; TG, triglyceride; LPa, lipoprotein.

### 3.2 Associations between serum uric acid and PhA

In univariate analysis, age, duration, Lpa, skeletal muscle mass (SMM), BMI, PBF, visceral fat area (VFA), TG, and UA were significantly associated with the phase angle (p <0.001) (**Table 2**).The b and corresponding 95% CIs for the phase angle according to serum uric acid tertiles are summarized in **Table 3**. Overall, in the crude model and all adjusted models (Models I–II), the phase angle increased as the tertiles of serum uric acid increased. In the fully adjusted model (Model II, adjustment for age, duration, Lpa, BMI,and WHR), the adjusted b for participants in the two tertiles of serum uric acid were 0.29 (95% CI: 0.08–0.5) and 0.41 (95% CI:0.19–0.62), respectively, compared with those in tertile 1.

**Table 2:** Association of phase angle and measured indicators.

Variables	β (95% CI)	*p*-Value
Age (years)	−0.027 (−0.033, −0.021)	<0.001
Duration (years)	−0.029 (−0.043, −0.014)	<0.001
TC (mmol/L)	0.079 (−0.004, 0.162)	0.0617
HbA1c (%)	−0.038 (-0.094, 0.018)	0.1862
Lpa (g/L)	−0.001 (−0.001, 0)	<0.001
FPG (mmol/L)	0.018 (-0.047, 0.084)	0.5774
FCP (nmol/L)	0.258 (−0.016, 0.533)	0.0651
SMM (kg)	0.08 (0.066, 0.094)	<0.001
BMI (kg/m^2^)	0.059 (0.03, 0.088)	<0.001
PBF (%)	−0.034 (−0.048, −0.021)	<0.001
WHR	1.266 (−0.495, 3.028)	0.1578
VFA (cm^2^)	−0.003 (−0.006, 0)	0.0324
TG (mmol/L)	0.106 (0.061, 0.151)	<0.001
UA (mmol/L)	0.002 (0.001, 0.003)	<0.001
sex: female vs male	−0.782 (−0.964, −0.599)	<0.001

CI, confidence interval; TC, total cholesterol; HbA1c, glycosylated hemoglobin; FPG, fasting plasma glucose; FCP, fasting C-peptide; SMM, skeletal muscle mass; BMI, body mass index; PBF, percent body fat; WHR, waist-to-hip ratio; VFA, visceral fat area; TG, triglyceride; LPa, lipoprotein; UA, uric acid.

**Table 3:** Association between serum uric acid and phase angle.

Variable	N	Crude model β (95% CI)	*p*-Value		Model I β (95% CI)	*p*-Value	Model II β (95% CI)		*p*-Value
UA	200	0.002 (0.001–0.003)	0.001		0.001 (0–0.002)	0.003	0.001 (0–0.002)		0.036
UA. T1	66	0 (Ref)			0 (Ref)		0 (Ref)		
UA. T2	67	0.32 (0.08–0.56)	0.011		0.29 (0.08–0.5)	0.008	0.26 (0.05–0.46)		0.016
UA. T3	67	0.53 (0.29–0.77)	<0.001		0.41 (0.19–0.62)	<0.001	0.32 (0.11–0.54)		0.004
P for trend			<0.001			<0.001			0.004

Crude Model: unadjusted.

Model I: adjust for age + duration.

Model II: adjust for age + duration+ Lpa + BMI + WHR.

N, number; UA, uric acid; CI, confidence interval.

### 3.3 Nonlinear relationship between serum uric acid and PhA

**Figure 2:**
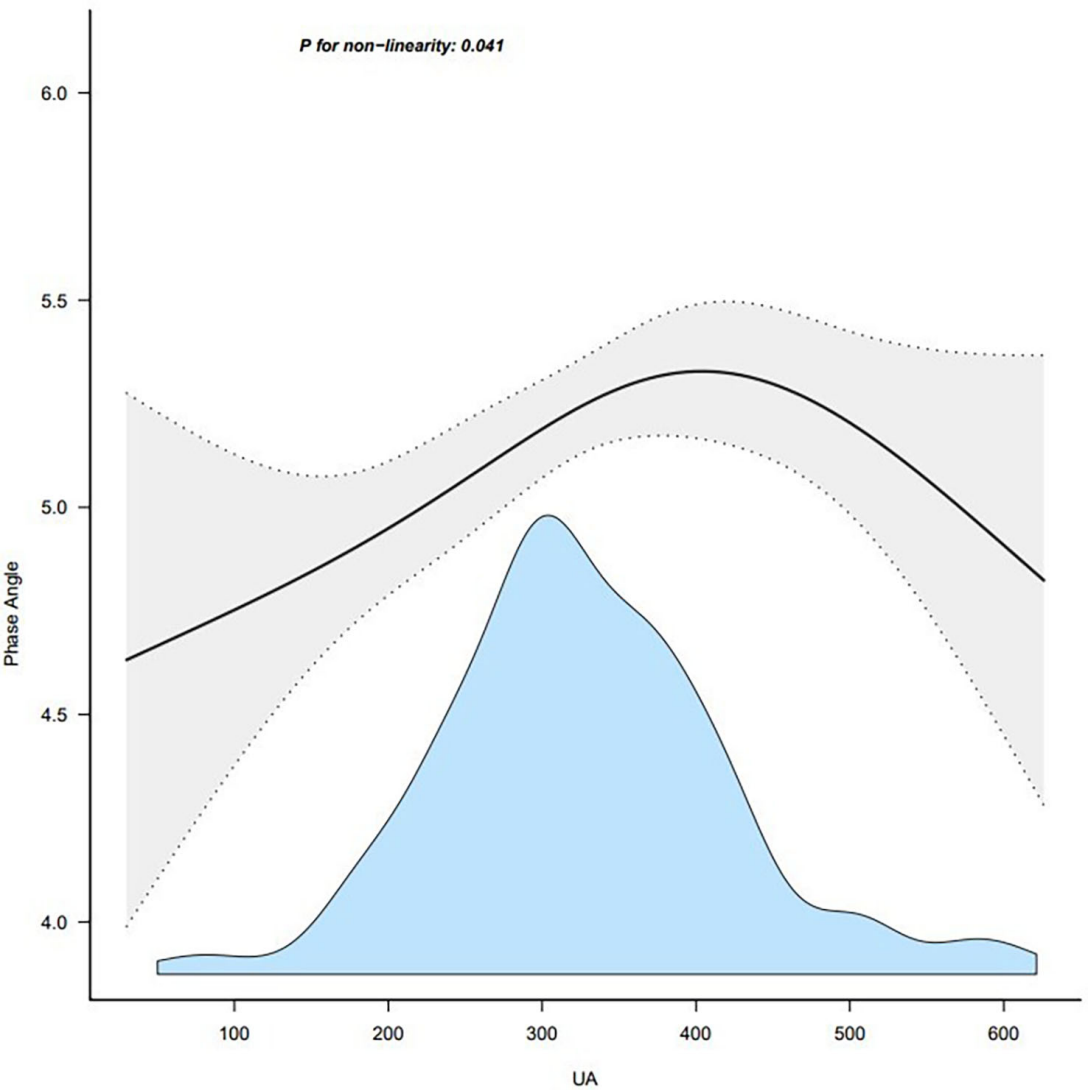
Association between UA and phase angle. Note: adjust for age + duration + body mass index. UA, uric acid.

We observed a nonlinear dose-response relationship between serum uric acid and PhA after adjusting for some covariates (**Figure 2**). Within a certain range, PhA progressively increased with serum uric acid levels, and the effect stopped beyond a certain threshold.

### 3.4 Sensitive analysis

**Figure 3:**
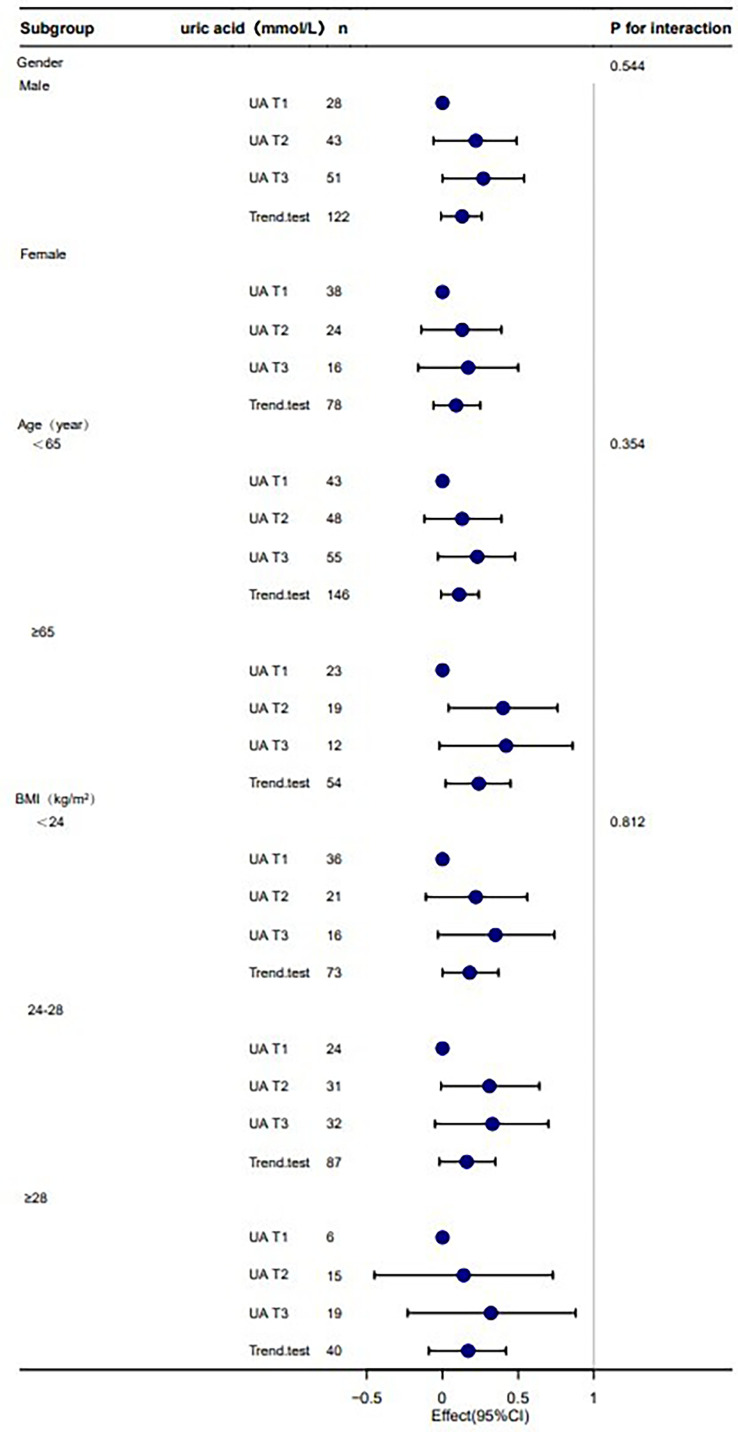
Stratification analysis on the association between UA and phase angle. Note: adjust for age + duration + BMI + Lpa + WHR. UA, uric acid; T, tertile; n number; BMI, body mass index.

The stratified analyses were performed to examine whether the association between serum uric acid and phase angle was stable among different subgroups. None of the variables, including gender (female and male), age (<65 years and ≥65 years), and BMI (<24 kg/m2, 24–28 kg/m2, ≥28 kg/m2), significantly affected the association between phase angle and serum uric acid (all P for interaction >0.05) (**Table 4**; **Figure 3**).

**Table 4:** Subgroup analysis between serum uric acid and phase angle.

Subgroups	N	UA T1 β (95%CI)/N	UA T2 β (95%CI)/N	UA T3 β (95%CI)/N	P for interaction
Sex					0.544
Male	122	0 (Ref)/28	0.22 (−0.06–0.49)/43	0.27 (0–0.54)/51	
Female	78	0(Ref)/38	0.13 (−0.14–0.39)/24	0.17 (−0.16–0.5)/16	
Age (years)					0.354
<65	146	0 (Ref)/43	0.13 (−0.12–0.39)/48	0.23(−0.03–0.48)/55	
≥65	54	0 (Ref)/23	0.4 (0.04–0.76)/19	0.42(−0.02–0.86)/12	
BMI (kg/m^2^)					0.812
<24	73	0 (Ref)/36	0.22 (−0.11–0.56)/21	0.35(−0.03–0.74)/16	
24–28	87	0 (Ref)/24	0.31 (−0.01–0.64)/31	0.33 (−0.05–0.7)/32	
≥28	40	0 (Ref)/6	0.14 (−0.45–0.73)/15	0.32(−0.23–0.88)/19	

adjust for age + duration + Lpa + BMI + WHR.

UA, uric acid; N, number; T, tertile; BMI, body mass index; CI, confidence interval.

## 4 Discussion

In this retrospective cross-sectional study, serum uric acid was independently associated with PhA. We observed a positive association between serum uric acid and PhA in patients with T2DM. The association was reliable and independent of essential covariates and confounders. To the best of our knowledge, this is the first report of an association between serum uric acid and PhA. Furthermore, the changing trend of the effective value at different serum uric acid levels was non-equidistant, which suggested that the association between serum uric acid and PhA was likely to be nonlinear. However, the effect of serum uric acid on PhA is significantly different in patients with T2DM when serum uric acid is below or above the threshold. According to the data in **Figure 1**, there is a threshold effect. Considering the clinical implications, the threshold could be inferred to be 420 ummol/L, which is also the threshold for hyperuricemia.

In numerous aspects, serum UA appears to be a two-faced marker of health and disease (14). The main cause of gout is hyperuricemia, which results in an elevated level of uric acid in the blood, whereas during peritoneal dialysis treatment, high uric acid is a positive prognostic factor (15). Other authors have previously concluded that BIA-derived phase angles can be associated with frailty and that phase angle values can be interpreted as a global marker of nutrition in aging (16). The phase angle is suggested to be an index of nutritional status (17), even better than anthropometric measurements or serum markers (18), which decrease with worsening of the nutritional status. On the one hand, phase angle increases as uric acid increases within a certain range, which may suggest uric acid could reflect nutrition. On the other hand, after exceeding a certain threshold of its concentrations, the metabolite can have a negative effect. Some studies have separately measured some variables of nutritional status and concluded that high UA levels are an indicator of better nutritional status, as evidenced by the positive association between UA levels and nutritional status variables. Furthermore, low concentrations of UA are considered a consequence of poor protein intake and the presence of malnutrition (19). Indeed, protein-rich diets tend to contain large quantities of purines (20), and higher uric acid concentrations may represent better nutritional status in the ESRD population. Some studies found a U- or J-shaped trend relationship between various outcomes and UA in different settings (21, 22). This could play a part in the observed different uric acid levels in the UA and PhA relationship because higher urate concentrations are associated with an inflammation status, which in turn might offset the potential beneficial effects of urate. From our experiment, we may be able to explain the relationship between uric acid and phase angle in a clinically meaningful way: before a certain threshold, uric acid can exist as a nutritional indicator and an antioxidant, and above a certain threshold, uric acid is harmful as an oxidant that accelerates damage.

To our best knowledge, this is the first study that explores the association between serum uric acid and PhA in T2DM patients. Phase angle will also be more likely elevated when serum uric acid is elevated, due to the ability of urate to indicate nutrition status. The results were, however, stable across the stratified subgroup analysis that was performed. As the UA increased, the PhA first increased and then decreased. It is interesting to consider the clinical implications of these results. Taking the clinical application and **Figure 1** information into consideration, we could set the threshold at 420 mmol/L.

Several shortcomings of the present study should be acknowledged. The study is limited by its cross-sectional design, which does not enable it to draw definite causal relationships. The number of participants was not very large. Insulin estimation was done in these subjects, and the effect of insulin on the kidney urate transporter 1 (URAT1) may be a confounder. In addition, bioelectrical impedance analysis was used to measure body composition; however, it has limitations. Finally, the present study included only the Chinese population with T2DM, so the results might not be representative of all patients with diabetes mellitus. However, it is worth mentioning that the interplay between serum uric acid and other factors has not yet been explored. Rethinking UA as a laboratory marker of nutritional status would require changing the dietary guidelines for subjects with T2DM.

## 5 Conclusion

There was a nonlinear relationship between serum uric acid and PhA in T2DM patients, and within a certain range, PhA progressively increased with serum uric acid raising, and the effect stopped beyond a certain threshold.

## Data availability statement

The datasets presented in this article are not readily available because the research is still ongoing. Requests to access the datasets should be directed to YH, hyz1932sun@163.com.

## Ethics statement

The studies involving human participants were reviewed and approved by the Ethics Review Board of the Zhongda Hospital Southeast University. Written informed consent for participation was not required for this study in accordance with the national legislation and the institutional requirements.

## Author contributions

HJ and JL designed this study. YH collected the clinical data and wrote the report. All authors contributed to the article and approved the submitted version.
